# Advancing pediatric antimicrobial stewardship: Has pharmacodynamic dosing for gram-negative infections taken effect?

**DOI:** 10.1017/ash.2021.199

**Published:** 2021-12-10

**Authors:** Lauren M. Puckett, Jason G. Newland, Jennifer E. Girotto

**Affiliations:** 1 University of Connecticut School of Pharmacy Storrs, Connecticut; 2 Connecticut Children’s, Hartford, Connecticut; 3 Washington University School of Medicine, St Louis, Missouri; 4 Antimicrobial Stewardship Program, Saint Louis Children’s Hospital, St Louis, Missouri

## Abstract

**Objective:**

To characterize pharmacodynamic dosing strategies used at children’s hospitals using a national survey.

**Design:**

Survey.

**Setting:**

Children’s hospitals.

**Participants:**

Volunteer sample of antimicrobial stewardship program (ASP) respondents.

**Methods:**

A nationwide survey was conducted to gain greater insight into the current adoption of nontraditional dosing methods and monitoring of select β-lactam and fluoroquinolone antibiotics used to treat serious gram-negative infections in pediatric populations. The survey was performed through the Sharing Antimicrobial Reports for Pediatric Stewardship (SHARPS) Collaborative.

**Results:**

Of the 75 children’s hospitals that responded, 68% of programs reported adoption of pharmacodynamically optimized dosing using prolonged β-lactam infusions and 35% using continuous β-lactam infusions, although use was infrequent. Factors including routine MIC monitoring and formal postgraduate training and board certification of ASP pharmacists were associated with increased utilization of pharmacodynamic dosing. In addition, 60% of programs reported using pharmacodynamically optimized ciprofloxacin and 14% reported using pharmacodynamically optimized levofloxacin. Only 20% of programs monitored β-lactam levels; they commonly cited lack of published guidance, practitioner experience, and laboratomory support as reasons for lack of utilization. Less physician time dedicated to ASP programs was associated with lower adoption of optimized dosing.

**Conclusions:**

Use of pharmacodynamic dosing through prolonged and continuous infusions of β-lactams have not yet been routinely adopted at children’s hospitals. Further guidance from trials and literature are needed to continue to guide pediatric pharmacodynamic dosing efforts. Children’s hospitals should utilize these data to compare practices and to prioritize further research and education efforts.

Antibiotic-resistant bacteria account for >2 million illnesses and 23,000 deaths yearly in the United States.^1^ A significant increase in multidrug-resistant (MDR) bacterial infections has occurred among pediatric patients in the past 20 years.^2,3^ Although newer antibiotic agents are available, they frequently come with a burden of cross resistance and lack pediatric efficacy and safety data on approval, which often limits their use in children with gram-negative infections.

An underutilized method for antimicrobial agent optimization is pharmacodynamic dosing modification, including prolonged and continuous infusion of β-lactams. These modalities have the potential to suppress development of antibiotic resistance and they have been shown to improve outcomes in adult patients.^4–10^ Utilizing prolonged or continuous infusion allows adequate time above the minimum inhibitory concentration (MIC) for the unbound antibiotic (*f*T>MIC) needed for bactericidal activity, as demonstrated in both adult and pediatric studies.^4–18^ In these analyses, the standard dosing of β-lactams and fluoroquinolones used to treat gram-negative infections is often reported to be inadequate due to the increasing MIC of gram-negative bacteria.^19–21^ Although a few cases using these approaches in children have been reported, the extent of adoption of these dosing strategies throughout pediatric practice is unknown.^22,23^ A critical need exists to assess current practices employed at children’s hospitals to serve as a foundation for improvement initiatives.

In this study, we characterized pharmacodynamic dosing strategies used at children’s hospitals by surveying the Sharing Antimicrobial Reports for Pediatric Stewardship (SHARPS) Collaborative. The assessment of β-lactam and fluoroquinolone nontraditional dosing strategies, limitations in dosing, as well as hospital demographics and pharmacist and physician dedication to antimicrobial stewardship (ASP) services, will guide future research.

## Methods

This study was reviewed and approved by the Institutional Review Board of University of Connecticut. Informed consent was obtained from participants in the study prior to survey completion. The survey was created in Qualtrics version June 2020 software (Qualtrics, Provo, UT) and included 35 multiple-choice and short-answer questions about dosing; use of pharmacodynamically optimized dosing; utilization of factors like MIC, susceptible-dose-dependent (SDD) defined MIC; and dosing based on severity of infection (Supplementary Table 1 online). Frequencies were defined as follows: always (>95%), frequently (51%–95%), sometimes (5%–50%), and rarely (<5%). Hospital and personnel demographics were collected, and associations were evaluated for the following factors: hospital characteristics, physician and pharmacist full-time equivalent (FTE) dedicated to ASP, and pharmacist training. Factors limiting utilization were also assessed. Survey questions were designed by the research team and were validated by a focus group of pediatric pharmacists prior to distribution.

The survey was distributed through the SHARPS Collaborative listserv, an international group of >750 individuals, primarily consisting of pediatric infectious diseases physicians and pharmacists within the United States. E-mail requests to participate in the study were distributed starting February 2020, and 3 reminder e-mails were sent before the survey closed June 6, 2020. Participants could skip any question they were not comfortable answering. A $25 gift card was used to incentivize survey completion. Only the first completed survey from each institution was included. Results were generalized by determining response rate and distribution. Frequency and descriptive statistics were used to characterize survey responses.

## Results

### Demographic characteristics

Responses were received from 75 institutions; 57% were from freestanding children’s hospitals. Among these hospitals, 47% had >200 beds, 33% had 101–200 beds, and 20% had 51–100 beds. Respondents primarily included pharmacists (81%) and physicians (16%). The median FTE for ASP physicians was 0.3 (interquartile range [IQR], 0.2–0.6) and the median FTE for ASP pharmacists was 1 FTE (IQR, 0.5–1).

### Pharmacist training and certifications

Pharmacist specialized training varied among programs. Overall, 85% of ASP pharmacists had completed a postgraduate year 1 (PGY1) and 67% had completed a PGY2 training program. A pediatric-focused PGY2 was the most commonly completed program (54%), followed by infectious diseases programs (32%) and pediatric infectious diseases programs (14%). Also, 13% of ASP pharmacists had completed other training, including Making a Difference in Infectious Diseases (MAD-ID) and Society of Infectious Disease Pharmacists (SIDP) antimicrobial stewardship certifications. Only 5% of ASP pharmacists had not completed any postgraduate training. Most ASP pharmacists had a board certification (69%), including BPS board-certified pharmacotherapy specialist (BCPS) (27%), BPS board-certified pediatric pharmacy specialist (BCPPS) (22%), BPS board-certified infectious diseases pharmacist (BCIDP) (11%), BCPS/BCIDP (16%), BCPS/BCPPS (11%), BCPS/BCPPS/BCIDP (4%), BCPPS/BCIDP (2%), and other (7%).

### Pharmacodynamic dosing

#### β-lactams

Among 75 hospitals, 62 (83%) provided information regarding dosing of β-lactam antibiotics. Of those, 85% used prolonged infusion (PI) and 35% used continuous infusion (CI) of a β-lactam. Respondents reported their PI use as frequently (7%), sometimes (31%), and rarely (31%). Fewer hospitals utilized CI (i.e., 25% sometimes and 34% rarely). Situations when respondents used PI or CI for β-lactams included history of MDR organisms (40%), cystic fibrosis exacerbations (33%), central nervous system infections (15%), and sepsis (8%). Other situations included endocarditis, critically ill children, modified dosing for outpatient parenteral antibiotics, and osteomyelitis. Antibiotics utilized for PI and CI included piperacillin-tazobactam (57% reported using it for PI and 20% for CI), meropenem (57% reported using it for PI and 11% for CI), cefepime (43% reported using it for PI and 8% for CI), and ceftazidime (31% reported using it for PI and 13% for CI). Ceftriaxone was also used for PI at 1 institution.

#### Fluoroquinolones

Fluoroquinolones are optimized by ensuring the appropriate total drug exposure or area under the curve. Previous Monte Carlo simulations have demonstrated that, for children aged 5–14 years, current dosing recommendations may not achieve a high probability of adequate pharmacodynamic targets for resistant organisms.^21,24^ In this survey, we applied a scenario-based question for a documented gram-negative sepsis in a 6-year-old child with normal renal function and asked which dose of ciprofloxacin and levofloxacin they would recommend, respectively. Dosing recommendations were reported by 58 (77%) of the 75 respondents for both ciprofloxacin and levofloxacin. Ciprofloxacin 30 mg/kg/day IV was the most commonly employed: 60% responded that they would divide every 8 hours and 5% would divide every 12 hours. In addition, 26% responded that they would utilize ciprofloxacin 10 mg/kg/day IV divided every 12 hours. For levofloxacin, 74% would utilize 10 mg/kg/day; 14% would utilize higher doses of 14 mg/kg/day divided twice daily and 3% would utilize 20 mg/kg/day divided twice daily. Finally, 7% of those who would use ciprofloxacin and 9% of those who would use levofloxacin stated that they would not use a fluoroquinolone for this scenario.

### Barriers to utilization of pharmacodynamic dosing

Survey respondents were asked to provide factors that discouraged them from utilizing pharmacodynamically optimized dosing, and they were able to select multiple answers. Lack of clear guidance on appropriate β-lactam serum levels was the most frequently indicated reason (48%), followed by lack of experience (36%), unreliable IV access (29%), lack of published data (24%), cost (20%), and lack of physician support (9%).

### Utilization of MIC, susceptible dose-dependent markers, and resistance markers

MIC was routinely monitored by 60% of responding programs. Thirty-five percent of programs routinely reported SDD. Routine monitoring of resistance-related gene markers [eg, extended-spectrum β-lactamases (ESBL), *Klebsiella*-producing carbapenemase (KPC)] was reported by 63% of responding programs. Overall, a moderate-to-low prevalence of gram-negative bacteria with borderline-susceptible MICs was described. In those 45 programs that monitor MIC, cefepime use for an MIC of >2 μg/mL, nonsusceptible for both Enterobacterales and *Pseudomonas aeruginosa*, was only reported as frequently used by 2%, as sometimes used by 53%, and as rarely used by 45%. Piperacillin-tazobactam resistance (i.e., MIC >16/4 μg/mL for Enterobacterales and *P. aeruginosa*) was reported as sometimes used by 76% and as rarely used by 24%. Meropenem use for MIC >1 μg/mL, which is nonsusceptible for Enterobacterales, was reported as sometimes used by 27% and as rarely used by 73%.

Also, 26 programs (35%) reported utilization and routine reporting of SDD. Of those 26 programs that reported SDD, 25% would use cefepime for organisms with MICs in this range with almost equal respondents using conventional dosing (n = 9) or prolonged infusions (n = 8).

Previously, Clinical and Laboratory Standards Institute (CLSI) recommended not performing screening or confirmatory testing for ESBL organisms but instead relying on lower MIC break points. Since then, there has been significant clinical debate regarding whether it is reasonable to treat susceptible organisms with an identified ESBL with select cephalosporins, specifically cefepime. In addition, 47 hospitals responded their comfort using cefepime for an ESBL-positive organism with low MIC: 55% suggested case-by-case evaluation, 36% would not use cefepime, and 9% were comfortable using cefepime if the organism was susceptible. Examples provided for case-by-case use included cystitis or urinary tract infection without bacteremia (15%), nonseptic patients (8%), tracheitis (4%), low-inoculum infections (4%), and use dependent on duration, treatment, and organisms (4%).

Survey participants were asked if they would adjust dose based on 1 or more of the following factors: MIC, organisms reported as SDD, or severity of infection. Moreover, 72% of programs reported that they would adjust antibiotic dosage based on severity of infection; 52% reported that they would adjust antibiotic dosage based on MIC; and 29% would adjust antibiotic dosage for an SDD organism.

### Therapeutic drug monitoring for β-lactams

Very few programs (n = 16, 21%) reported the use of therapeutic drug monitoring (TDM) for β-lactams. Of those that reported TDM, 1 hospital reported always using TDM, 6 hospitals reported using TDM sometimes, and 9 hospitals reported that they rarely utilized TDM. Of the programs that reported using TDM for β-lactams, most occurred when pharmacokinetics were expected to be altered: 33% used TDM when patients had altered renal function, 27% used it in patients receiving extracorporeal membrane oxygenation, and 20% used it in cystic fibrosis patients. Other TDM uses included in cases in which there was uncertainty of dose (20%), those who did not respond to conventional regimens (20%), or in patients with either infections with confirmed MDR organisms, or those with infections with resistant organisms limiting treatment options. Among respondents, 17% provided a goal, including targets ranging from >50% time above MIC (T>MIC; n = 7) to 40%–50% T>MIC (n = 4) to 100% T>MIC (n = 2).

### Associations with dose modifications and pharmacokinetic/pharmacodynamic dosing

An evaluation was performed to determine associations between pharmacist or physician FTE and employing specific processes. Institutions with a larger physician FTE for ASP had higher frequencies of PI and CI use (Table 1). Physician FTE did not affect the use of TDM for β-lactams or routine monitoring of MIC. We detected no trends with pharmacist FTE in relation to use of modified infusion times or use of TDM (Table 1).

**Table 1.** Dedicated Physician Full Time Equivalent to ASP Activities in Relation to Use of Pharmacodynamic Dose Modification

Variable	Dedicated ASP Physician FTE, Median (IQR)	Dedicated ASP Pharmacist FTE, Median (IQR)
**Use of prolonged infusions**		
Frequently (n = 5)	0.5 (0.3–0.7)	1 (0.5–1)
Sometimes (n = 23)	0.5 (0.3–0.9)	1 (0.5–1)
Rarely (n = 21)	0.3 (0.2–0.5)	0.9 (0.5–1)
Never (n = 11)	0.3 (0.1–0.3)	1 (1–1)
**Use of continuous infusions**		
Sometimes (n = 14)	0.81 (0.3–1)	0
Rarely (n = 21)	0.3 (0.2–0.6)	1 (0.6–1)
Never (n = 24)	0.3 (0.2–0.4)	0.9 (0.5–1)
**TDM for β-Lactams**		
Always (n = 1)	0.25	1
Sometimes (n = 6)	0.3 (0.2–0.5)	0
Rarely (n = 9)	0.4 (0.3–0.7)	0.5 (0.4–0.6)
Never (n = 42)	0.3 (0.2–0.6)	1 (0.7–1.5)
**Performs routine MIC monitoring**		
Yes (n = 44)	0.3 (0.2–0.5)	1 (0.5–1)
No (n = 12)	0.3 (0.2–0.5)	1 (1–1)
**Dosing modifications performed based on factors**		
MIC (n = 38)	0.35 (0.3–0.6)	1 (0.5–1)
SDD (n = 21)	0.5 (0.3–0.8)	1 (0.5–1)
Severity (n = 53)	0.3 (0.2–0.5)	1 (0.5–1)

Note. ASP, antimicrobial stewardship program; FTE, full-time equivalent; IQR, interquartile range; TDM, therapeutic drug monitoring; MIC, minimum inhibitory concentration; SDD, susceptible dose dependent.

Overall usage of pharmacodynamic dosing (based on MIC, SDD, and severity of infection) was analyzed based on pharmacist training and board certifications (Table 2). Most hospitals reported that ASP pharmacists had received PGY1 training or beyond. Among them, most stated that they would adjust dose based upon MIC or severity of infection. Institutions with pediatric or pediatric infectious diseases–trained pharmacists responded similarly, noting that they would adjust dose based upon MIC, SDD, and severity. The only category in which >50% of infectious diseases trained pharmacists reported preference for dose adjustment was severity of infection. Notably, institutions with no board-certified pharmacists stated that they would adjust dose for SDD, whereas institutions with BCPPS and BCIDP certified pharmacists reported adjusting dose for MIC and severity of infection. Lastly, those with BCPS-certified pharmacists reported that they would modify dose only for infection severity.

**Table 2.**
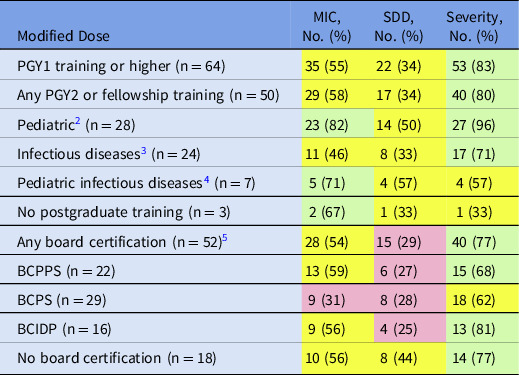
Pharmacist Training in Relation to Modified Dosing Regimens Based on Patient Factors

Note. MIC, minimum inhibitory concentration; SDD, susceptible dose dependent; PGY, postgraduate year of training; BCPPS, board-certified pediatric pharmacy specialist; BCPS, board-certified pharmacotherapy specialist; BCIDP, board-certified infectious diseases pharmacist. PGY1 or higher includes pharmacists that noted training of PGY1, PGY2 (any type), fellowship or any combination of those training modalities. Pediatrics training includes (pediatrics (PGY2 or fellowship). Pharmacist infectious diseases training includes infectious disease (PGY2 or fellowship) and pediatric infectious disease (PGY2 or Fellowship). Pediatric infectious diseases training includes only pediatric infectious disease (PGY2 or fellowship). Some pharmacists have multiple trainings, so numbers of specific trainings are higher than overall number with board certification. Red: 0 to < 33%; Yellow 33% to < 66%; Green >/ = 66%

**Fig. 1.**
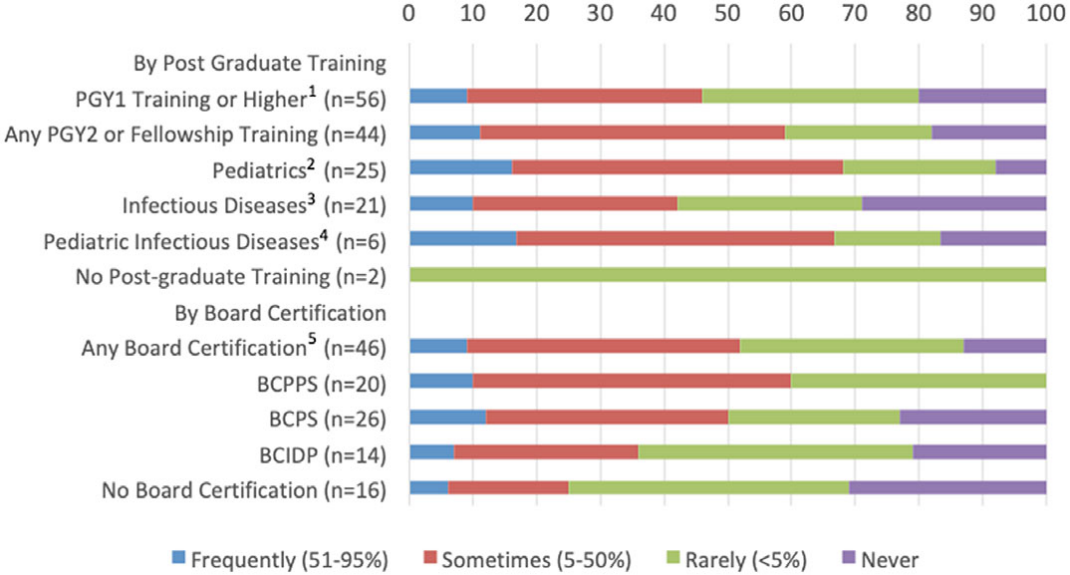
Relation of pharmacist training to frequency of use of prolonged infusions of β-lactams. Note. PGY, postgraduate year of training; BCPPS, board-certified pediatric pharmacy specialist; BCPS, board-certified pharmacotherapy specialist; BCIDP, board-certified infectious diseases pharmacist. (1) PGY1 or higher includes pharmacists that noted training of PGY1, PGY2 (any type), fellowship or any combination of those training modalities. (2) Pediatrics training includes pediatrics (PGY2 or fellowship). (3) Pharmacist infectious diseases training includes infectious disease (PGY2 or fellowship) and pediatric infectious disease (PGY2 or fellowship). (4) Pediatric infectious diseases training includes only pediatric infectious disease (PGY2 or fellowship). (5) Some pharmacists have multiple trainings, so numbers of specific trainings are higher than the overall number with board certification are higher than overall number with board certification.

**Fig. 2.**
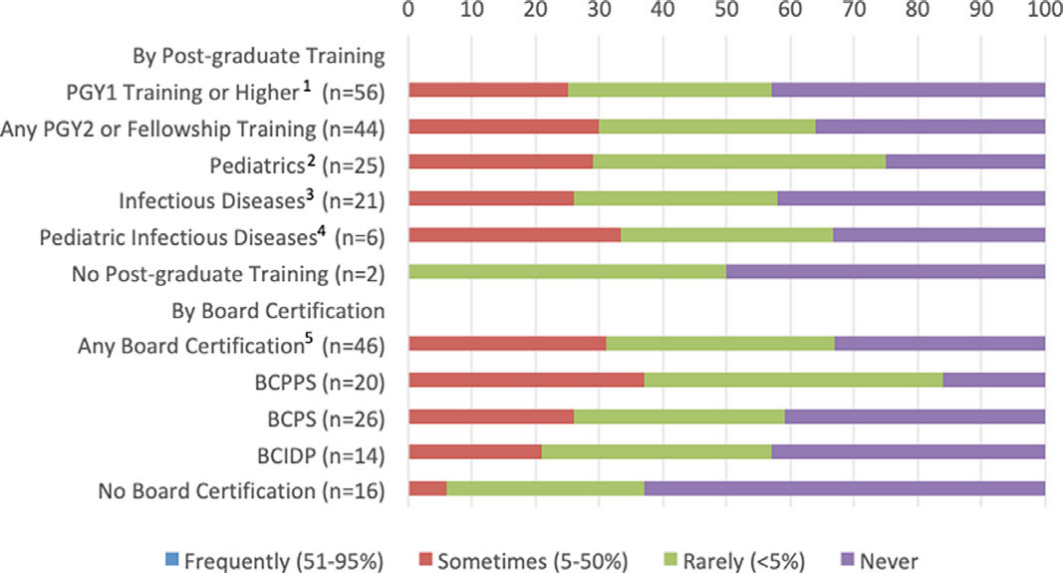
Relation of Pharmacist training to frequency of use of continuous infusions of β-lactams. Note. PGY, postgraduate year of training; BCPPS, board-certified pediatric pharmacy specialist; BCPS, board-certified pharmacotherapy specialist; BCIDP, board-certified infectious diseases pharmacist. (1) PGY1 or higher includes pharmacists that noted training of PGY1, PGY2 (any type), fellowship or any combination of those training modalities. (2) Pediatrics training includes pediatrics (PGY2 or fellowship). (3) Pharmacist infectious diseases training includes infectious disease (PGY2 or fellowship) and pediatric infectious disease (PGY2 or fellowship). (4) Pediatric infectious diseases training includes only pediatric infectious disease (PGY2 or fellowship). (5) Some pharmacists have multiple trainings, so numbers of specific trainings are higher than the overall number with board certification are higher than overall number with board certification.

Although programs with pharmacists who had been trained in pediatrics (92%) or pediatric infectious diseases (80%) reported using PI at least rarely, 29% of programs with infectious diseases–trained pharmacists reported never using this modality (Figs. 1 and 2). Institutions in which pharmacists had BCPPS certification had the highest reported use of PI or CI (100% reported using PI and 84% used CI) versus pharmacists with BCPS (77% used PI and 59% used CI) or BCIDP (79% used PI and 57% used CI). We also detected a significant association between routine MIC monitoring and the use of modified dosing regimens. Institutions that did not routinely monitor MIC utilized significantly less PI (*P* < .001) and CI (*P* = .01) than institutions that utilized routine monitoring.

## Discussion

Treatment of gram-negative infections in children continues to be challenging due to increasing MICs and prevalence of resistance. A recent meta-analysis showed an increase in MDR *P. aeruginosa* from 15.4% to 25% between 1999 and 2012.^25^ One potential method for suppressing resistance development is pharmacodynamic dosing.^4–7^ Although these methods have shown enhanced clinical response rates and improvement in surrogate markers of outcomes in adults, only a small amount of published information has shown similar outcomes in pediatric patients.^8–10,22,23,26–^ ^34^ Unfortunately, because pediatric patients have different pharmacokinetic parameters than adults, the specifics of dosing optimization cannot be simply extrapolated from adult models. Monte Carlo simulations have been recommended as one method to help optimize pediatric antibiotic dosing.^12^ Many of these simulations have found that traditional dosing of β-lactams was only predicted to provide bactericidal activity for many organisms with low MICs. Courter et al^20^ showed the potential for continuous and prolonged infusions to optimize pharmacodynamics of time-dependent antibiotics in children. Although traditional doses with standard intermittent infusions were unlikely to provide bactericidal activity against *P. aeruginosa* strains, use of higher dosing or PI or CI achieved 90% likelihood of bactericidal exposures for common β-lactam antibiotics.^20^

In this study, most children’s hospitals use PI of cefepime, piperacillin-tazobactam, and meropenem only in patients with known high MICs or unique pharmacokinetic parameters (e.g., cystic fibrosis). In comparison, most rarely or never used CI for β-lactams. Reasons for the not using PI or CI were lack of guidelines, lack of experience, and insufficient patient venous access.

Although the pediatric Surviving Sepsis guidelines recommend the use of PI and CI in treating pediatric patients, they do not provide any guidance regarding dosing or monitoring.^35^ To address these barriers, studies assessing benefits and outcomes in pediatric patients are needed.

Monte Carlo simulations have also been performed to evaluate and optimize fluoroquinolone dosing in children.^21,24^ In simulations, dosages of ciprofloxacin at 30–40 mg/kg/day every 12 hours were needed to achieve high probability of meeting pharmacodynamic targets.^24^ For levofloxacin, the specific dosage needed for optimal bactericidal activity against gram-negative organisms is uncertain. A Courter et al^21^ model suggested that in children 5–14 years, even doses of levofloxacin 8 mg/kg every 12 hours only achieved pharmacodynamic targets in 90% of patients for gram-positive bacteria with an MIC ≤ 1 mg/L.^21^ Given that gram-negative bacteria require higher pharmacodynamic targets, optimal activity would only be seen at even lower MICs.

Based on survey data, pharmacodynamically optimized dosing of ciprofloxacin was more commonly utilized than optimized dosing of levofloxacin (by 65% and 17% of respondents, respectively). Interestingly, almost 10% of respondents said that they would not use a fluoroquinolone to treat gram-negative sepsis. These responses could possibly reflect different interpretations of the survey question, the fact that fluoroquinolones are not a first-line–recommended agent in pediatric sepsis, or limited literature or previous experience with suboptimal outcomes.

The TDM of β-lactams has been used sporadically in pediatrics, and use will likely increase thanks to new recommendations in the Pediatric Surviving Sepsis Campaign International Guidelines.^35^ In this survey, TDM was reportedly used at 20% of institutions, with lack of published guidelines, testing and laboratory support identified as common barriers. Responses also reflected an overall lack of experience and confidence with employing TDM; few respondents provided an opinion on appropriate therapeutic goals for β-lactam monitoring. For this technology to see wider utilization, more literature, guidance, and timely processing are needed.

Debate continues regarding the use of certain cephalosporin antibiotics to treat ESBL-positive organisms.^36^ Data from adults studies suggest that outcomes are closely related to the MIC of the infecting organism and that clinical failure significantly increases with increasing MIC.^37–39^ No specific pediatric data are available on this topic, making it uncertain whether lowering the MIC break points is sufficient to ensure successful outcomes with susceptible ESBL-positive organisms. This cautious outlook seems to be shared by survey respondents, who reported using cephalosporin antibiotics only on a case-by-case basis (63%) or never (36%). Example scenarios in which respondents would use a cephalosporin included low-inoculum infections and instances in which an infection is exposed to high levels of antibiotic for an extended period (e.g., cystitis without bacteremia treated with renally eliminated antibiotics).

The implementation of the SDD category by the CLSI has allowed for retained utilization of important antimicrobials in adult patients by providing specific dosing regimens to use with higher MICs.^40^ Caution should be taken when extrapolating CLSI recommended doses to pediatric patients due to differing pharmacokinetic parameters in children. and such dosing should be evaluated specifically at the SDD MIC.^20,41^ Concern about extrapolating these standards to pediatric patients was shared by most survey respondents; only 29% of programs reported dose modification with SDD MIC organisms.

This study included a large number of participating hospitals, in part due to the utilization of the SHARPS Collaborative. As expected, most responding institutions were freestanding children’s hospitals with >100 beds. Most survey responders were ASP pharmacists. Surveyed hospitals were supported by a median of 1 FTE ASP pharmacist with some level of postgraduate training.

Programs employing a pharmacist with pediatric training, either in pediatrics or pediatric infectious diseases, were more frequent users of PI and CI β-lactams, and they performed more dose modification based on MIC and infection severity. Although this finding is likely multifactorial, it is probably related to the children’s hospitals hiring well-trained pharmacists to manage critically ill children and pharmacists with pediatric training better understanding children’s unique pharmacokinetics.

In this study, most reporting hospitals were ≥100 beds and met the recommended ASP pharmacist FTE percentage with a median of 1 FTE.^42^ These factors may be why pharmacist FTE was not associated with any observed differences in responses. Physician FTE was lower, at a median of 0.3 (IQR, 0.2–0.6). Importantly, sites that reported lower ASP physician FTE were less likely to participate in dose optimization strategies, perhaps because the physician is not given sufficient time to actively assist and champion the ASP.^42^

This study has several limitations. Data collection was from respondents entering information based upon their practice. Bias may have been introduced into the data because only 1 staff member responded to the survey questions, and different staff may have had different responses based on their experiences. This study may also have been affected by sampling bias; only hospitals that were part of the SHARPS Collaborative listserv received the survey. Most responding institutions were also freestanding children’s hospitals, potentially limiting the generalizability of these findings to other institution types. Because response was voluntary, institutions that elected to participate in this survey may inherently place higher priority on antimicrobial dosing initiatives, which may have introduced further bias to the analysis.

Overall, variation exists in the optimization of dosing antimicrobials for pediatric patients in an era of increasing resistance. Further guidance by national and international groups is needed to continue to guide and increase pharmacodynamic dosing of children.

## Supplementary material

For supplementary material accompanying this paper visit https://doi.org/10.1017/ash.2021.199. click here to view supplementary material
