# Supplementary material for: Advancing pediatric antimicrobial stewardship: Has pharmacodynamic dosing for gram-negative infections taken effect?
Source: Antimicrob Steward Healthc Epidemiol. 2021 Dec 10;1(1):e61. doi: 10.1017/ash.2021.199 (PMC9495429; doi:10.1017/ash.2021.199)
Supplement: Supplementary file 1 [file S2732494X21001996sup001.docx]

**Supplementary Table 1.** Survey Questions

| **Demographics** | | |
| --- | --- | --- |
| 1. | Hospital Name |  |
| 2. | Number of pediatric beds at hospital? | - <50 - 51–100 - 101–200 - >200 |
| 3. | Are you a free-standing children’s hospital? | - Yes - No |
| 4. | Which best describes your role? | - Physician - Pharmacist - Other (free-text option) |
| 5. | Please describe training of the ID/ASP pharmacist(s) at your hospital (select all that apply). | - Pediatrics (PGY2 or fellowship) - Infectious disease (PGY2 or fellowship) - Pediatric infectious disease (PGY2 or fellowship) - PGY1 trained only - No postdoctoral training - Other (free text option) |
| 6. | Is the hospital’s ID/ASP pharmacist(s) board certified? | - Yes - No |
| 7. | If you marked “yes” for question 6, Please describe training of the ID/ASP pharmacist(s) board certification (select all that apply). | - BCPS - BCIDP - BCPPS - Other |
| 8. | How many physician FTEs are dedicated to pediatric antimicrobial stewardship? | (Free text) |
| 9. | How many pharmacist FTEs are dedicated to pediatric antimicrobial stewardship? | (Free text) |
| **For the clinical questions for each antibiotic below please answer the to the best of your ability. For the following antibiotic questions (10–13), please assume dosing for children (eg. 2–10 years of age) with normal renal function.** | | |
| 10. | Do you ever use prolonged infusion β-lactams at your institution? | - Always (>95%) - Frequently (50%–95%) - Sometimes (5%–51%) - Rarely (<5%) - Never |
| 11. | If ‘yes’, which agents? (select all that apply) | - Ceftriaxone - Ceftazidime - Cefepime - Piperacillin-tazobactam - Meropenem - Other (free text) |
| 12. | Do you ever use continuous infusion β-lactams at your institution? | - Always (>95%) - Frequently (50%–95%) - Sometimes (5%–51%) - Rarely (<5%) - Never |
| 13. | If “yes,” which agents? (select all that apply) | - Ceftriaxone - Ceftazidime - Cefepime - Piperacillin-tazobactam - Meropenem - Other (free text) |
| *If you answered anything except "never" on questions 10 or 12, please answer question 14.* | | |
| 14. | Are there disease states that you are more likely to use prolonged or continuous infusions to treat? | - Sepsis - History of multidrug-resistant organisms - Cystic fibrosis - Central nervous system infection - Other (free text) |
| For questions 15 and 16 please provide the empiric dose you would use for a child (eg, 6 years old) with documented gram-negative sepsis and normal renal function | | |
| 15. | What ciprofloxacin dosing would you use in a patient with documented gram-negative sepsis? | - 10 mg/kg IV q8h - 10 mg/kg IV q12h - Other (Free text) |
| 16. | What levofloxacin dosing would you use in a patient with documented gram-negative sepsis? | - 10 mg/kg IV daily - 7 mg/kg IV q12h - Other (Free text) |
| 17. | Do you use therapeutic drug monitoring for β-lactam therapies? | - Always (>95%) - Frequently (50%–95%) - Sometimes (5%–51%) - Rarely (<5%) - Never |
| 18. | If you answered ‘frequently’ ‘sometimes’ or ‘rarely’, what are situations where you would monitor levels for β-lactams antibiotics? | - (Free text) - N/A |
| 19. | What therapeutic goal do you use for evaluation of β -lactam levels? | - 30-40% T>MIC - 40-50% T>MIC - >50% T>MIC - Other (free text) |
| 20. | Are there existing factors that deter your institution from utilizing pharmacodynamic dosing? If, yes, please select all that apply. | - No - IV line access or availability issues in patient - Lack of published data on safety or efficacy - Lack of physician support - Lack of experience - Lack of clear guidance on appropriate levels - Cost - Other (free text) |
| **Antimicrobial stewardship** | | |
| 21. | Does your institution routinely monitor MIC on isolated pathogens? | - Yes - No |
|  | *If you selected “yes” for question 21, please answer questions 22-24 below.* | |
| 22. | When reviewing MICs of your gram-negative bacteria in practice, do you frequently see cefepime MIC >2? | - Always (>95%) - Frequently (50%–95%) - Sometimes (5%–51%) - Rarely (<5%) |
| 23. | When reviewing MICs of your gram-negative bacteria in practice, do you frequently see a piperacillin-tazobactam MIC>16/4? | - Always (>95%) - Frequently (50%–95%) - Sometimes (5%–51%) - Rarely (<5%) |
| 24. | When reviewing MICs of your gram-negative bacteria in practice, do you frequently see a meropenem MIC >1? | - Always (>95%) - Frequently (50%–95%) - Sometimes (5%–51%) - Rarely (<5%) |
| 25. | Does your institution report ‘Susceptible-Dose-Dependent’ (SDD) MIC as defined by CLSI guidelines? | - Yes - No |
|  | *If you said “yes” to question 25, please answer question 26 and 27.* | |
| 26. | Does your institution use cefepime for treatment of infections that are SDD to cefepime in pediatric patients? | - Yes - No |
| 27. | How does your institution dose cefepime based on SDD MIC in pediatric patients? | - Standard dosing - Continuous infusion - Prolonged infusion |
| 28. | Does your institution include in the culture and sensitivity report if bacteria are positive for extended-spectrum β-lactamase (ESBL)? | - Yes - No |
|  | *If you answered "yes" to question 28, please answer question 29 below.* | |
| 29. | Do you feel comfortable using a cephalosporin (eg, cefepime) for ESBL(+) organisms with low MICs? | - Yes - No - Case by case (free text) |
| 30. | Do you dose based on organism MIC, SDD, or severity of infection? | - Yes - No |
